# Two Pictures

**Published:** 1878-11

**Authors:** 


### TWO PICTURES.

On the 12th day of October last, Judge Williams of this city delivered an opinion in the case of Nathan T. Aikin vs. The State Board of Health, a portion of which is well worth reproduction.

The name of Aikin does not appear on the register of the physicians of the State, published under the auspices of the local and State Medical Societies. It would appear, however, that, having presented proofs to the Board of Health that he was possessed of a properly issued diploma in medicine, Aikin secured a license to practice, made out in regular form. Thereupon he proceeded to make known his special skill in various departments of medicine,by means of newspaper advertisements, which came finally to the notice of the Board. The plaintiff was thereupon notified by the Board of Health, that such advertisements would not be considered proper when put forward by their licenciates; and this remonstrance induced Aikin to submit to the Board a form of public notice or advertisement, which seems to have met with their approval. This the defendant proceeded to insert in the daily papers; but afterward, either emboldened by his success or stimulated by an urgent need of it, he proceeded to amplify this permitted advertisement till it became again obnoxious to the Board. Aikin was then given notice to appear before them and show cause why his license should not be revoked. He then proceeded by civil process to test the powers popularly supposed to be vested in the Board by the law which called it into existence. The action was in the form of a petition for an injunction from the court prohibiting the State Board of Health from depriving the plaintiff of his license to practice medicine. The case was well argued on both sides; elicited a great deal of comment from many of the laity as well as the professional gentlemen of the city ; and its decision is, as will be seen, decidedly in favor of the State authorities.

After reviewing the facts and the arguments, and quoting the clauses of the statute empowering the State Board to revoke certificates for cause, the learned judge said :—

The State Board was a corporation constituted expressly to have charge of medical practice and practitioners, and to exercise surveillance over the professional conduct of physicians, so as to exclude empirics and raise the standard of medical acquirements. Such a Board must of necessity be vested with large discretion. It ought to be so vested, and in the legitimate exercise of its discretion, ought not and could not be properly controlled by judicial tribunals. Its duties and position made it fit to judge of the professional conduct of physicians, and no power was given to any other body or officer to supervise it.

A physician might be guilty of unprofessional and dishonorable, and yet not of criminal conduct. It would have been a work of supererogation for the legislature to have given the State Board supervision over unprofessional conduct if that and criminal conduct had been synonomous. The term "unprofessional" was far wider than "criminal," and what was "unprofessional" conduct could only be determined by bringing the act to the professional criterion, and nobody could be better qualified to decide such questions than the State Board, five out of seven of whom were physicians themselves. "Unprofessional" conduct did not, therefore, necessarily involve criminal or immoral acts, but was such conduct as was inconsistent with the honorable practice of the profession, and, in judging of such conduct, the Board of Health had a wide discretion, which should not be interfered with by courts.

The general rule of law applicable to the point was, that equity would not interfere, by injunction, for the purpose of controlling the action of public officers, constituting inferior quasi-judicial tribunals on matters properly pertaining to their jurisdiction, nor would it review and correct errors in the proceedings of such officers, the proper remedy, if any, being at law, by writ of certiorari; and when they had exercised their decision, in good faith, and without any intention of oppressing or injuring private persons, an injunction would not be allowed against their action.

But, independent of the exercise of discretion, it appeared, as a matter of ıact, that the advertisements of Aikin were unprofessional, for a large number of the most eminent physicians in the city had testified to that effect, and some had added that such advertisements were false.

Another objection by Aikin was, that the law creating the State Board was unconstitutional, depriving some physicians of their property without due process of law. It was, therefore, necessary to determine whether a license to practice a profession was a constitutional privilege or property. Any man was at liberty to choose his own profession, but he could only practice it on the terms imposed by the law, and the law could impose such terms on any profession or employment as the legislature in its discretion deemed for the best interests of the community. The law had always sought to fill the learned professions with learned, upright, and honest men; and, though it had sometimes failed, the attempt was in the right direction, and for that purpose it had hedged law and medicine round with licenses. Men who had the property and lives of others especially intrusted to their keeping, ought to be men of skill and learning. More than that, it was of the utmost importance that all dishonor and dishonesty should be expelled from the learned professions, and the tendency of legislation had always been to effect that result. It had been expressly decided that the right to practice law was not a constitutional right, derived from the law of nature, but a mere creation of the statute, and a license only conferred a statutory right, subject to the control of the legislature. It was not property, nor even a contract between the legislature and attorney.

In no proper sense could the words "property" and "contract" be applied to the right to practice medicine. Such right was not descendible from its possessor to his heir, could not be bought or sold, and might be lost by misconduct or immorality on the part of the practitioner. It was not necessary, in order to constitute uniformity in the law, that it should bear equally upon all citizens of the State, but only that it should bear equally upon all who stood in the same relation to it, upon all who were under substantially the same facts. A physician who had practiced ten years would, by that practice, acquire a knowledge of local diseases and their treatment not to be attained by a stranger to the region, however extensive might be his reading. Every rule must to a certain extent, be arbitrary, and a Court would hesitate to declare a law unreasonable because it applied to some under one state of facts and not to others dissimilarly situated. And a Court would decline to set aside positive enactments of the legislature merely on the ground that in their opinion the law was unreasonable.

But, as the right to practice medicine was a mere statutory privilege, subject to be changed at any time by the legislature, and did not rise to the dignity of a contract or of property, there was no reason why such a privilege should not be denied to one man and extended to another in the discretion of the legislature. The objections to the law for want of uniformity, therefore, were not well taken.

The motion for injunction would accordingly be overruled.

Knowing the incompleteness and the actual legal deficiencies of the law under which the Board of Health was organized, we cannot fail to regard it as a matter for congratulation, that the courts have consented to construe its provisions, not by its letter which is defective, but by its spirit, which is calculated to awaken the sympathies of all professional men in good standing. Whether the Board of Health shall stand or fall, whether its powers be curtailed or enlarged by a future legislature, we find abundant cause for congratulation that one of our eminent judges has laid down a valuable precedent in an opinion which reflects credit upon himself and the profession to which he belongs. It is an opinion written in that large and comprehensive view of the status of the professional man, which recognizes the fact that, while all men have inherent rights and privileges in the matter of barter and trade, the best interest of the community requires that law and medicine should be hedged about with such licenses, that only the learned and the upright shall be privileged by recognized membership. Surely the State Board of Health need not be reminded that this decision, justly in their favor, is due far more to the influence exerted by the general character of the professional men in the country upon the community at large, than upon any act or acts of theirs, as a corporate body. The decision is in general terms, upon professional conduct as viewed by "five physicians," out of the seven members of the Board.

For the Board itself, which may be said to be still on trial before the public and the profession, no verdict is found. The victory of the Board—and it is a victory—has been won rather by the honorable record of the mass of the profession, than by the five men referred to in the decision.

Very soon after its organization, we announced in these pages that we were unwilling to hamper the first steps of the Board by any adverse criticism. Though often tempted to comment in an unfavorable manner upon certain of their procedures, we have steadfastly refrained from uttering a word which would embarrass or cripple them in the performance of their duties. Nor shall we do so now. We wish for them the largest degree of usefulness which it is possble for them to achieve. We believe that, indeed, they can scarcely fail to do more in each succeeding year, than they have done in the past year of their labors. We believe also that the composition of the Board—its union of homœopathic, eclectic and regular practitioners—has been admitted to be no bar to the faithful execution of its task. But we have a stern duty to perform, as independent journalists, and from that duty we shall not shrink. We propose to point out the glaring inconsistency in the gross results accomplished by their action, which has been fretting the conscience of the physicians of this city and State ever since January, 1878.

When Dr. Aikin went out of court, non-suited, he might have purchased a daily newspaper published in this city. That newspaper, a few months ago, was engaged in what the editors thought was a crusade against the medical schools of Chicago, on the ground that the latter should adopt a higher standard of education in science. And the editors of this sheet were at the same time receiving money from the publication of the flaming advertisements, the *ipsissima verba* of which we copy from the *Chicago Times*, and commend to Dr. Aikin's consideration:—

DR. JAMES, LOCK HOSPITAL, 204 WASHINGTON ST., COR. FRANKLIN, CHICAGO.—Chartered by the State of Illinois for the express purpose of giving immediate relief in all cases of private, chronic and urinary diseases in all their complicated forms. It is well known Dr. James has stood at the head of the profession for the past thirty years. Age and experience are all important. Seminal weakness, night losses by dreams, pimples on the face, lost manhood, can positively be cured. Ladies wanting the most delicate attention, call or write. Pleasant home for patients. A book for the million—Marriage Guide—which tells you all about these diseases who should marry, why not; 10 cents to pay postage. Dr. James has fifty rooms and parlors. You see no one but the Doctor. Office hours, 9 a.m. to 7 p.m.; Sunday, 10 to 12. Dr. James is 60 years of age. Stammering and stuttering cured, or no pay.

CURE YOURSELF.—French Specific Mixture, guaranteed to cure radically, diseases of certain secret and delicate nature in either sex or condition. Price $1. Full directions with each bottle. Sent by express. Sold only by E. L. Stahl, cor. Van Buren and Fifth ave., Chicago.

DR. A. G. OLIN'S PRIVATE HOSPITAL, 203 SOUTH CLARK ST., CHICAGO.—Everybody, from Atlantic to Pacific, has heard of Dr. Olin's skill in treating chronic and sexual diseases of men and women, spermatorrhœa, sexual debility, impotency, nervousness, seminal emissions, loss of memory from self-abuse or other causes, cured permanently. "Guide to Health," 64 pages, two 3-cent stamps—large work, choice information of special interest to both sexes, 50 cents. Reliable female pills and rubber goods at office. Special care, with board, for ladies during confinement.

DR. HENDERSON, 171 EAST MADISON STREET, CHICAGO, ILL. *A regular graduate in medicine. Over ten years' practice.*

Treats all forms of Chronic, Nervous, and Private Diseases, Spermatorrhœa, Sexual Debility, etc. Guarantees his best attention and treatment. Charges reasonable. Medicines furnished. Patients at a distance treated by letter. Consultation free and confidential—call or write. Illustrated BOOK and circulars sent sealed for two 3c stamps—free at office. 8 a. m. to 7 p. m. Sundays, 2 to 4.

NO CURE! NO PAY! DR. KEAN, No. 173 South Clark Street, Chicago, is still treating all Private, Nervous, Chronic and Special diseases, Spermatorrhœa, Impotency (sexual incapacity), Female diseases, etc. Consultation personally or by letter free. Green book, illustrated, 50 cents. Dr. Kean is the only physician in the city that warrants cure or no pay All languages spoken.

The authors of these advertisements are practicing medicine here in Chicago, in the manner set forth with great clearness by themselves. Some of them are said to be operating under assumed names, and to have had their "hospitals" and other institutions incorporated under the general law of incorporation of the State —the very source from which the State Board of Health acquires all its powers and emoluments. They may have become exempt by virtue of the clause which excludes all from the operation of the licensing law who have been practicing medicine in this State for more than ten years. And they may also be not exempt— who knows? The bare fact remains that, after almost one year of the existence of the State Board, these men, by the aid of the *Chicago Times*, are flinging the spume of the brothel and the filth of the sexually depraved, upon the breakfast tables of a large proportion of the news-reading public.

We know what may be said in answer to all this. Rome was not built in a day. The Board of Health cannot correct all evils at once, and do they not well if they correct a few? Even admitting all that may be said in regard to their limited powers, we cannot ignore these facts. There is a love of fair play and fair dealing inherent in the Anglo-Saxon, that cannot complacently contemplate the spectacle of the incautious youths in the city and the more experienced peripatetic peddlers of nostrums in the country, arrested in their career by the strong arm of the law, which yet is paralyzed in the presence of the hoary scoundrels, rooted in the heart of almost all our larger towns, strong mainly in the wealth accumulated in the very sewers of society, debauching the public morals in the public prints, and defying all the restraints imposed in the name of decency.

Till this great ulcer is healed or hidden from the public gaze, the professional men of this city cannot applaud with enthusiasm the operations by which the Board of Health may seek to remove an insignificant blemish upon the visage of the profession displayed to the public view.

